# Characterization and assessment of barnacle larval settlement-inducing activity of extracellular polymeric substances isolated from marine biofilm bacteria

**DOI:** 10.1038/s41598-019-54294-9

**Published:** 2019-11-28

**Authors:** Aboobucker Siddik, Sathianeson Satheesh

**Affiliations:** Department of Marine Biology, Faculty of Marine Sciences, King Abdulaziz University, Jeddah, Saudi Arabia

**Keywords:** Microbial ecology, Marine biology

## Abstract

Extracellular polymeric substances (EPSs) are the hydrated gelatinous matrix produced by microorganisms for attachment in a biofilm environment. In this study, the compositional variation between EPSs of three marine biofilm bacteria (*Pseudoalteromonas shioyasakiensis, Vibrio harveyi* and *Planomicrobium* sp.) were analysed by GC-MS, ^1^H NMR, FT-IR and XRD and SEM. The ecological significance of exopolymers was assessed *in vivo* using marine model organism barnacle larvae for their settlement-inducing activity. Chemical analysis revealed the presence of glycan fucosylated oligosaccharides, tetraose, trisaccharides, iso-B-Pentasaccharides, sialyllactose, oligomannose, galacto-*N*-biose, difucosyl-para-lacto-*N*-neohexaose, 3′-sialyl *N*-acetyllactosamine and isoglobotriaose-β-*N*(Acetyl)-Propargyl in all extracted EPSs. Bioassay results indicated that treatment of the barnacle larvae with EPSs from three bacterial strains enhanced settlement on substrates. In conclusion, this study highlighted the role of water-soluble EPSs in the invertebrate larval settlement on artificial materials.

## Introduction

Persistence of marine benthic population has been determined by the larval settlement and metamorphosis of marine invertebrates^1^. Marine invertebrates begin settlement by responding to biological or chemical stimuli or cues^2^. Biological and chemical cues such as microbial biofilm, conspecific cues, extracellular polymers, free fatty acids, peptides, crustose coralline algae, neurotransmitter compounds, quorum sensing compounds and tetrabromopyrrole have been reported to induce settlement of larval forms of marine organisms^3–11^. Among the cues, extracellular polymeric substances (EPSs) or exopolymers are the gelatinous matrix produced by microorganisms for their linkage or attachment in a biofilm environment^12^. High molecular weight polysaccharides are the major constituents of exopolymers produced by microorganisms^13,14^. The other components of EPSs include proteins, amyloid fibrils, nucleic acids and uronic acids^12,15,16^.

The composition and structure of exopolymers vary between bacterial species, and is highly dependent on the nutrient source and environmental conditions^13,17^. The detailed analysis and characterization of exopolymers is difficult. Least explained in the literature is the composition of polymeric substances and their role in larval settlement and metamorphosis^18,19^. The majority of exopolymers derived from marine microbes are natural heteropolysaccharides, composed of different repeat monomers^20^. The characterization of exopolymers produced by marine biofilm bacteria is important for understanding the interaction between larvae and biofilms^21^.

Experimental evidence has substantially proved that exopolymers from different microbial origin have different effects (induction or inhibition) against marine invertebrate larvae^10,22,23^. For instance, Morse and Morse^24^ reported that the sulphated polysaccharides present in algal cell surfaces induced the settlement of coral *Agaricia humilis* larvae. Also, the high molecular weight extracellular polymers of diatom species *Achnanthes* sp. and *Nitzschia constricta* induced the larval settlement of *Hydroides elegans*, whereas the low molecular weight exopolymers induced a low number of larval settlement^5^. Inversely, extracellular polymers derived from the marine biofilm bacteria *Roseobacter litoralis* have no settlement-inducing effect on *H. elegans*^25^. The exopolymers of biofilms, diatom species and *Pseudomonas aeruginosa* induced settlement and metamorphosis in *Amphibalanus amphitrite* larvae^26,27^. Conversely, exopolymers derived from *Bacillus pumilus* and *Citrobacter freundii* showed no effect on settlement and metamorphosis of *A. amphitrite* larvae^27^.

In this study, an attempt was made to assess the settlement-inducing activity of EPSs produced by marine biofilm-forming bacteria isolated from artificial materials submerged in the central Red Sea. Exopolymers isolated from biofilm bacterial strains were tested against the larvae of barnacle *A. amphitrite*. Like other marine invertebrates, *A. amphitrite* has greater ecological significance among rocky intertidal habitats and it is also commonly used as a model organism in antifouling studies^28–30^. Specifically, the following questions were addressed in this study: (1) do the exopolymers induce settlement and metamorphosis of *A. amphitrite* larvae? and (2) is the chemical composition similar in all the three exopolymers isolated from *P. shioyasakiensis*, *V. harveyi* and *Planomicrobium* sp.? Results obtained in this study will further advance our knowledge of the role of marine biofilms in larval settlement.

## Results

### Larval toxicity and settlement assay

**Figure 1:**
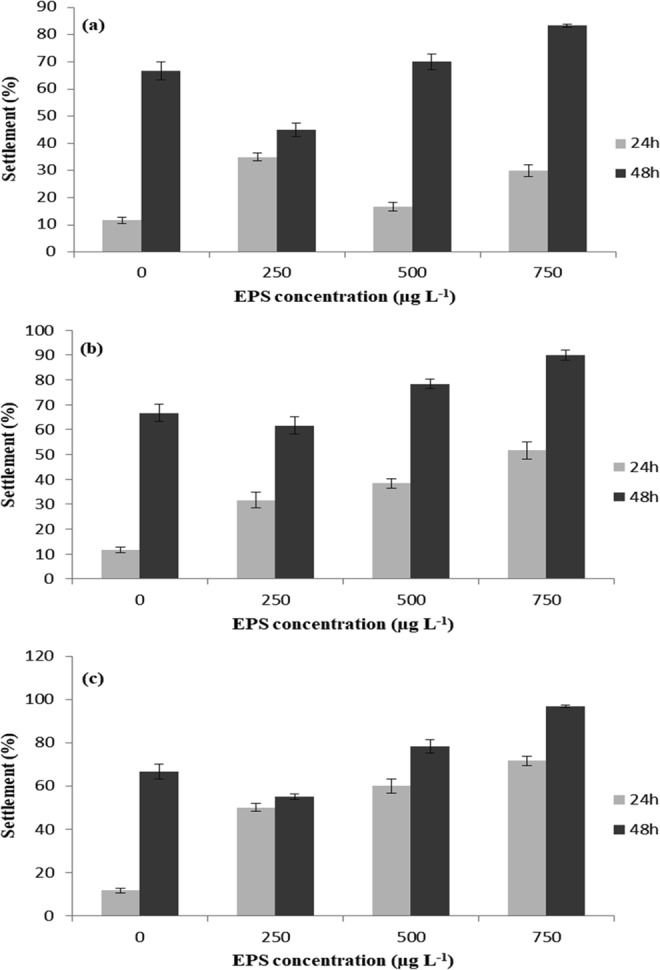
Barnacle larval settlement (mean ± SD, n = 6) in response to different concentrations of EPS isolated from (**a**) *Pseudoalteromonas shioyasakiensis* EPS-Psh, (**b**) *Vibrio harveyi* EPS-Vha and (**c**) *Planomicrobium* sp. EPS-Pla.

Exopolymers obtained from *P. shioyasakiensis*, *V. harveyi* and *Planomicrobium* sp. showed no toxicity towards barnacle nauplii after 24 and 48 h. Mortality was not observed during the treatment. Further, EPSs enhanced the settlement of barnacle larvae. The larval settlement of *A. amphitrite* increased considerably with increasing concentrations of EPSs (Fig. 1a–c). In the control group, 11.66% of cyprid settlement was observed after 24 h. After 48 h, 66.66% of cyprid settlement was noticed in the control group. Moreover, the maximum percentage of settlement (96.66%) was observed in cyprids treated with 750 µg l^−1^ concentrations of EPS-Pla. The larval groups treated with EPS-Psh and EPS-Vha showed a maximum cyprid settlement of 83.33% and 90% (750 µg l^−1^) respectively. The EC_50_ values of EPS-Psh, EPS-Vha and EPS-Pla were 498.29, 494.36 and 500.09 µg l^−1^ respectively. Two-way analysis of variance (ANOVA) revealed a significant variation in the settlement of larvae treated with different concentrations of EPS-Vha and EPS-Pla (Table 1). The settlement of larvae treated with different concentrations of EPS-Psh did not show significant difference between treatments. However, the interaction between concentration and observation time revealed significant variation (Table 1).

**Table 1:** Two-way ANOVA results for the cyprid settlement treated with different concentrations of EPS.

Source of Variation	EPS-Psh			EPS-Vha		EPS-Pla	
	df	F	P	F	P	F	P
Time	1	48.6205	0.000	27.377	0.000	14.784	0.0004
Concentration	3	1.739	0.174	3.15	0.0352	8.507	0.0001
Interaction	3	3.182	0.034	0.444	0.722	2.476	0.0751
Within	40						
Total	47						

^*^P < 0.05 = significant.

### Fluorescence microscopy imaging of biofilm

**Figure 2:**
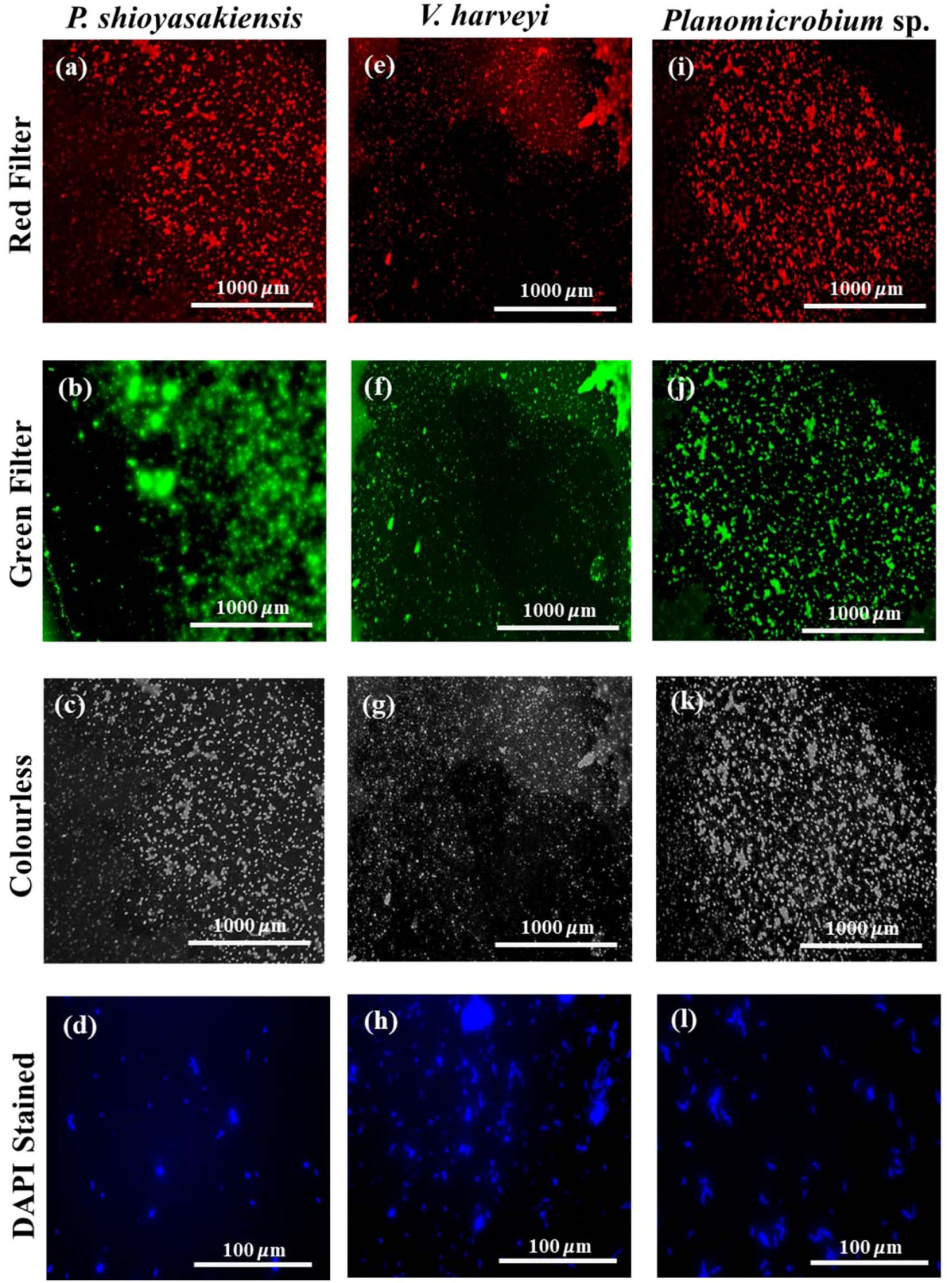
Fluorescence microscopy visualization of biofilms developed on 24-well cell culture plates (**a–c**) *Pseudoalteromonas shioyasakiensis*, (**e–g**) *Vibrio harveyi* and (**i–k**) *Planomicrobium* sp. DAPI staining of biofilm bacterial cells (scale 100 *μ*m): (**d**) *P. shioyasakiensis*, (**h**) *V. harveyi* and (**l**) *Planomicrobium* sp.

Microscopy images confirmed the biofilm-forming ability of the three bacterial strains under laboratory conditions. The direct fluorescence microscopy imaging of 24-h-grown biofilms is shown in Fig. 2. The biofilm images were captured using different filters: red filter (Fig. 2a–c), green filter (Fig. 2e–g), colourless (visible) filter (Fig. 2i–k) and DAPI filter (Fig. 2d,h,l). The biofilm mats formed by bacterial cells were visible at the scale of 1000 µm (4x objective). An enlarged view of biofilm bacterial cells captured using 40x objective (100 µm scale) is presented in Supplementary Fig. 2. DAPI stain clearly showed the presence of bacterial cells in biofilm matrix.

### ^1^H NMR analysis

**Figure 3:**
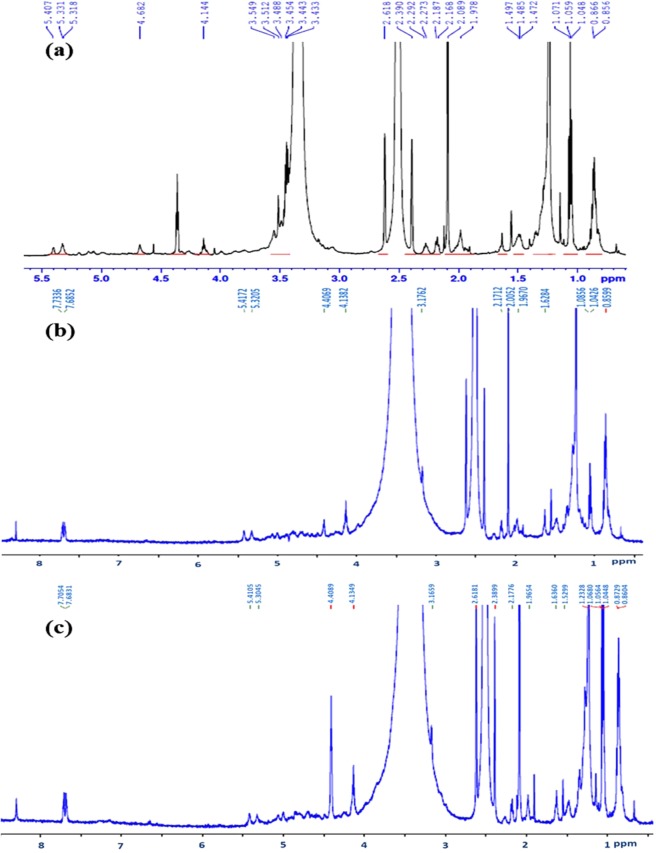
Proton (^1^H) NMR spectroscopy analysis of EPS isolated from (**a**) *Pseudoalteromonas shioyasakiensis*, (**b**) *Vibrio harveyi* and (**c**) *Planomicrobium* sp.

The 600 MHz proton NMR spectra of EPSs in DMSO are shown in Fig. 3a,b,c. The ^1^H NMR spectral peaks of EPS-Psh were observed at δ 0.85, 0.86, 1.04–1.07 ppm, alkyl region 1.47–1.49 ppm, 2.08–2.61 ppm, ring proton region 3.43–3.54, 4.14 ppm, anomeric proton region 4.68 and 5.31–5.40 ppm. EPS-Vha and EPS-Pla have mass shift at δ 0.86 ppm, 1.04–1.08 ppm, alkyl region 2.08–2.17 ppm, anomeric proton region 4.13–4.40 ppm, 5.30–5.41 ppm and 7.68–7.70 ppm. The spectral peaks of EPS-Vha and EPS-Pla were similar to each other.

### GC-MS analysis

**Figure 4:**
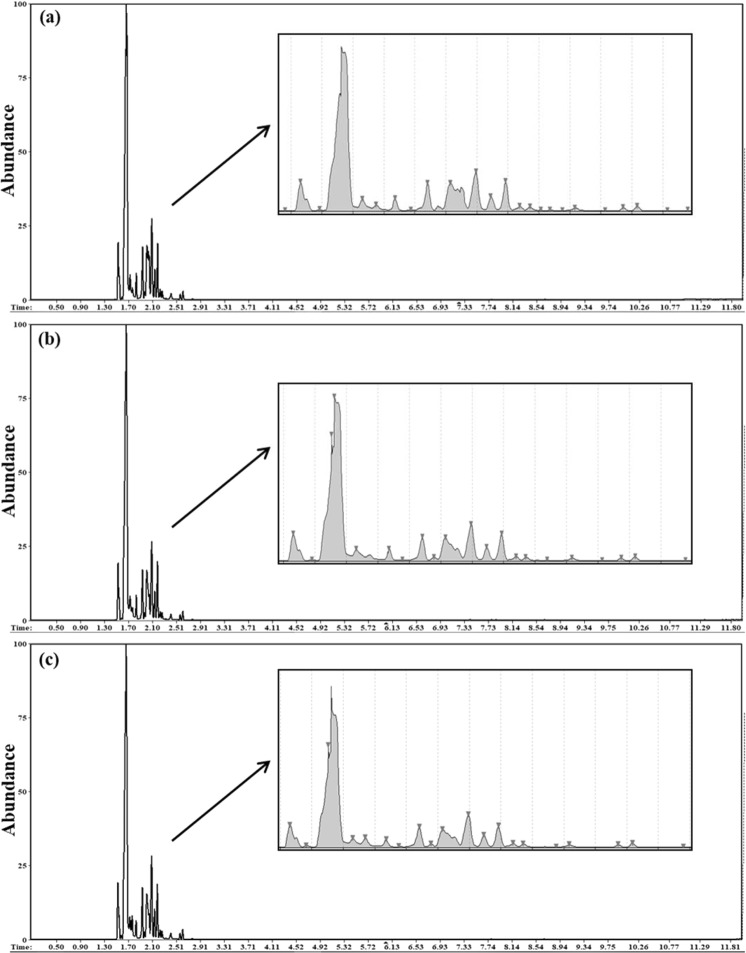
GC-MS analysis of EPS isolated from (**a**) *Pseudoalteromonas shioyasakiensis*, (**b**) *Vibrio harveyi* and (**c**) *Planomicrobium* sp.

**Figure 5:**
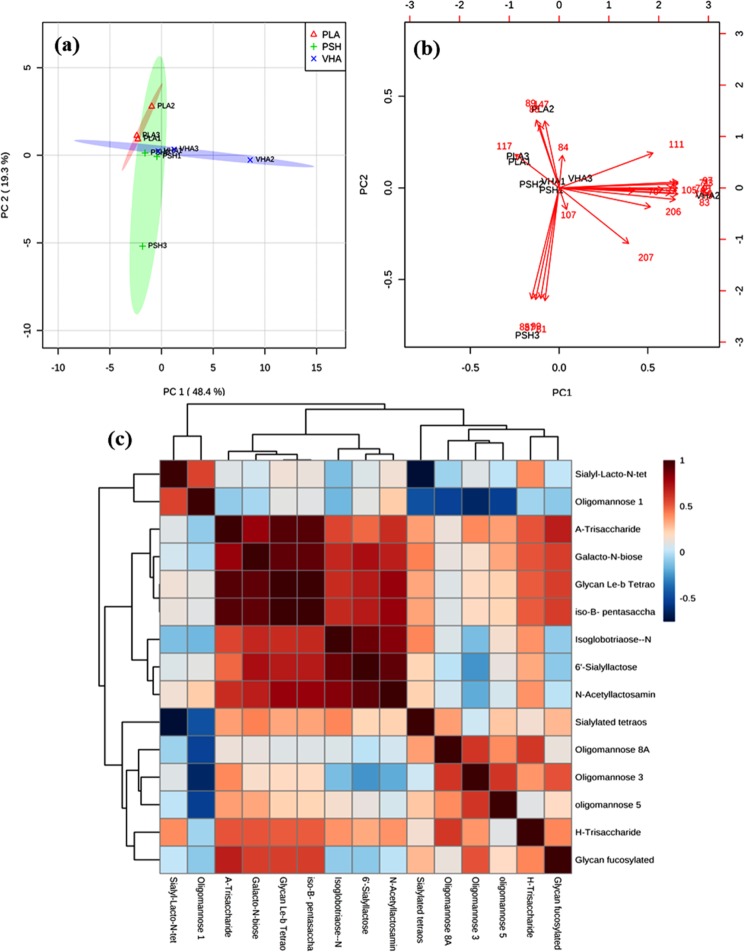
Principal component analysis of EPS isolated from *Pseudoalteromonas shioyasakiensis*, *Vibrio harveyi* and *Planomicrobium* sp. (**a**) PC1 vs PC2 2D scores plot and (**b**) PC1 vs PC2 biplots. (**c**) The correlation heat map analysis of EPS-Psh, EPS-Vha and EPS-Pla.

The total carbohydrate and protein content of the EPSs are shown in Table 2. Glycosyl composition analysis of EPSs produced by *P. shioyasakiensis*, *V. harveyi* and *Planomicrobium* sp. (Fig. 4a,b,c) showed that the EPS consists of tetraose, trisaccharides, iso-B-Pentasaccharides, sialyllactose, oligomannose, galacto-*N*-biose, glycan fucosylated oligosaccharide, 3′-sialyl Lewis X, difucosyl-para-lacto-*N*-neohexaose, 3′-sialyl *N*-acetyllactosamine and isoglobotriaose-β-*N*(Acetyl)-Propargyl (Tables 3–5). The variations in glycosyl composition among three EPSs (EPS-Psh, EPS-Vha and EPS-Pla) were determined using principal component analysis (PCA), a non-targeted multivariate statistical tool. The PCA scree plot of the accumulated variance explained by PC1-PC7 showed that PC1 contributed 48.4% to the total variance observed (Fig. 5a). The other significant components are given by PC2-PC7 with percentage contributions of 19.3%, 13.9%, 10.6%, 3.3%, 2.5% and 1.6% respectively. PC1-PC7 accounted for 99.7% of the accumulated variance of the samples. The PCA score plot and biplot of the principal components PC1 and PC2 showed that exopolymers of EPS-Psh, EPS-Vha and EPS-Pla were categorized in the same cluster except VHA2 and PSH3 (Fig. 5a,b). The correlation heatmap analysis showed that Glycan Le-b tetraose and iso-B-Pentasaccharides are closely related to each other (Fig. 5c).

**Table 2:** Total carbohydrate and total protein content of EPS (µg mg^−1^).

EPS	Total carbohydrate content (µg mg^−1^)	Total protein content (µg mg^−1^)
EPS-Psh	130	45
EPS-Vha	88	38
EPS-Pla	111	64

**Table 3:** GC-MS analysis of glycosyl composition of exopolymers isolated from *Pseudoalteromonas shioyasakiensis* (EPS-Psh).

	Glycosyl composition	Target Ion [*m*/*z*]	Retention Time	Intensity
1	Glycan Le-b Tetraose	99	1.48	2912731
2	Difucosyl-para-lacto-*N*-neohexaose	74	1.53	1.90E + 08
3	6′-Sialyllactose	71	1.59	8506785
4	Sialyl-Lacto-*N*-tetraose	73	1.73	7.82E + 07
5	A-Trisaccharide	70	1.78	3.97E + 07
6	6′-Sialyllactose	71	1.84	7.96E + 07
7	*N*-acetyllactosamine	87	1.89	5159836
8	3′-Sialyl Lewis X	83	1.94	1.82E + 08
9	Glycan fucosylated oligosaccharide	79	2.01	1.82E + 08
10	H-Trisaccharide	74	2.1	2.54E + 08
11	iso-B-Pentasaccharide	97	2.14	9.45E + 07
12	Galacto-*N*-biose	84	2.19	1.93E + 08
13	iso-B-Pentasaccharide	97	2.24	2.98E + 07
14	Sialylated tetrose type 2	97	2.27	2.57E + 07
15	Unidentified	85	2.31	4837636
16	Isoglobotriaose-β-*N*(Acetyl)-Propargyl	71	2.34	5358300
17	Oligomannose 8 D1D3	99	2.38	3234973
18	Glycan fucosylated oligosaccharide	83	2.42	2.03E + 07
19	A-Type II Tetrasaccharide	95	2.51	3143567
20	Unknown compounds	91	2.57	2.18E + 07
21	Unknown compounds	91	2.62	3.10E + 07
22	3′-Sialyl Lewis X	82	2.72	1554738
23	Unidentified	91	2.78	4485566
24	A-Type II Tetrasaccharide	111	3.86	1236336
25	Oligomannose 8 A	161	7.79	3071352
26	Oligomannose 3 A	206	7.87	2074843
27	Oligomannose 5	107	8.06	2075195
28	Galacto-*N*-biose	207	8.69	4617695

**Table 4:** GC-MS analysis of glycosyl composition of exopolymers isolated from *Vibrio harveyi* (EPS-Psh).

	Glycosyl composition	Target Ion [*m*/*z*]	Retention Time	Intensity
1	Glycan Le-b Tetraose	99	1.48	1786271
2	Difucosyl-para-lacto-*N*-neohexaose	74	1.53	1.72E + 08
3	6′-Sialyllactose	71	1.59	7849301
4	3′-Sialyl-*N*-acetyllactosamine	107	1.65	8.13E + 08
5	6′-Sialyllactose	82	1.66	1.06E + 09
6	Oligomannose 6	73	1.73	7.40E + 07
7	A-Trisaccharide	70	1.84	7.65E + 07
8	iso-B-Pentasaccharide	73	1.88	5837818
9	3′-Sialyl Lewis X	83	1.94	1.52E + 08
10	6′-Sialyllactose	71	1.98	2.41E + 07
11	Glycan Le-b Tetraose	79	2.02	1.50E + 08
12	4β-Galactobiose	74	2.1	2.34E + 08
13	iso-B-Pentasaccharide	97	2.15	8.74E + 07
14	Galacto-*N*-biose	84	2.19	1.76E + 08
15	iso-B-Pentasaccharide	97	2.24	2.76E + 07
16	Sialylated tetraose type 2	97	2.27	2.42E + 07
17	Isoglobotriaose-β-*N*(Acetyl)-Propargyl	85	2.34	4713606
18	Glycan fucosylated oligosaccharide	83	2.42	1.98E + 07
19	Le-A Trisaccharide	70	2.51	2342311
20	Unidentified	91	2.57	1.81E + 07
21	Unidentified	91	2.62	2.81E + 07
22	6-Sialylgalactose	91	2.78	4008337
23	3′-Sialyl-*N*-acetyllactosamine	79	7.79	2578279
24	3′-Sialyl Lewis X	105	8.06	1655128
25	iso-B-Pentasaccharide	206	8.68	3367370

**Table 5:** GC-MS analysis of glycosyl composition of exopolymers isolated from *Planomicrobium* sp. (EPS-Psh).

	Glycosyl composition	Target Ion [*m*/*z*]	Retention Time	Intensity
1	Glycan Le-b Tetraose	99	1.48	1692712
2	Difucosyl-para-lacto-*N*-neohexaose	74	1.53	1.71E + 08
3	H-Trisaccharide	89	1.58	1.33E + 07
4	3′-Sialyl-*N*-acetyllactosamine	88	1.65	6.57E + 08
5	3′-Sialyl Lewis X	117	1.66	1.06E + 09
6	Sialyl-Lacto-*N*-tetraose	73	1.73	1.07E + 08
7	A-Trisaccharide	147	1.77	7.60E + 07
8	Glycan lacto-N-hexaose	70	1.84	8.10E + 07
9	Galacto-*N*-biose	73	1.88	6332569
10	3′-Sialyl Lewis X	83	1.94	1.64E + 08
11	iso-B-Pentasaccharide	71	1.98	2.68E + 07
12	Glycan Le-b Tetraose	71	2.02	1.32E + 08
13	Galacto-*N*-biose	77	2.1	2.62E + 08
14	Galacto-*N*-biose	97	2.15	9.96E + 07
15	3′-Sialyl Lewis X	84	2.19	1.75E + 08
16	6′-Sialyllactose	97	2.24	3.22E + 07
17	iso-B-Pentasaccharide	97	2.27	2.72E + 07
18	Glycan Le-Y Tetraose	84	2.31	4848386
19	Galacto-*N*-biose	71	2.34	5502553
20	Glycan fucosylated oligosaccharide	83	2.42	2.13E + 07
21	6′-Sialyllactose	125	2.52	2719762
22	Unidentified	91	2.57	2.18E + 07
23	A-Trisaccharide	91	2.62	3.34E + 07
24	3α,4β,3α-Galactotetraose	82	2.72	1653783
25	Not identified	91	2.78	4346030
26	Oligomannose 1 F	81	3.86	1072264
27	iso-B-Pentasaccharide	91	7.79	2131376
28	Monogalactosylated N glycan	207	7.86	1662071
29	Oligomannose 3 A	111	8.68	3543469
30	*N*-acetyllactosamine	206	11.51	3129848

### FT-IR analysis

**Figure 6:**
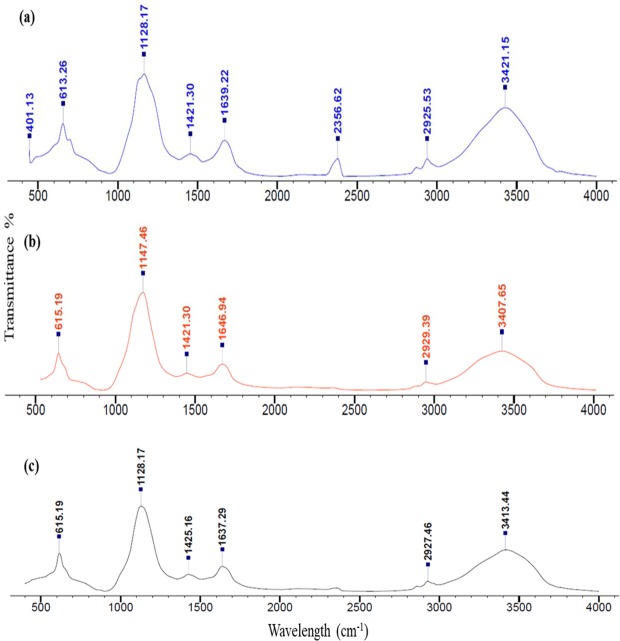
Fourier-transform infrared (FT-IR) spectroscopy analysis of EPS isolated from (**a**) *Pseudoalteromonas shioyasakiensis*, (**b**) *Vibrio harveyi* and (**c**) *Planomicrobium* sp.

The FT-IR spectra of exopolymers EPS-Psh, EPS-Vha, and EPS-Pla are shown in Fig. 6. The IR spectra of all the three exopolymers were similar and displayed a broad stretching peak at 3407–3414 cm^−1^ possibly due to the presence of a hydroxyl group. Peaks were also observed around 2926–2929 cm^−1^, 1637–1646 cm^−1^, 1421–1424 cm^−1^, 1127–1147 cm^−1^ and 500–650 cm^−1^. A small peak was observed around 2356 cm^−1^ in EPS-Psh, but not in EPS-Vha and EPS-Pla.

### X-ray diffraction analysis of EPS

**Figure 7:**
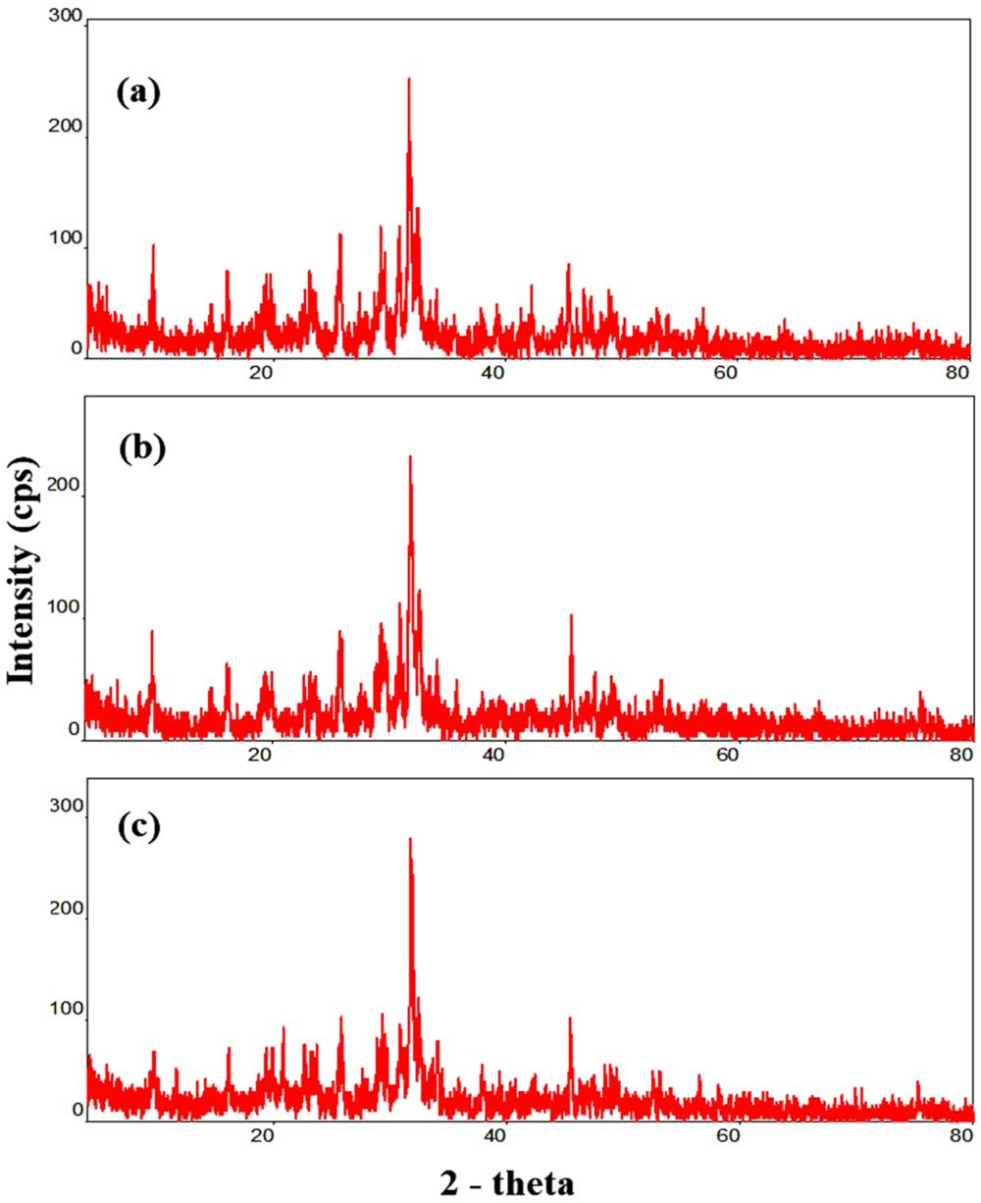
X-ray diffraction analysis of EPS isolated from (**a**) *Pseudoalteromonas shioyasakiensis*, (**b**) *Vibrio harveyi* and (**c**) *Planomicrobium* sp.

The XRD spectra (Fig. 7a–c) of three EPSs with their respective inter-planar spacings (d-spacings) indicate the crystalline nature of exopolymers (Fig. 7). The XRD spectral patterns of EPS are attributed to amorphous characteristics, with a crystalline phase. The crystalline index (CI_XRD_) of extracted EPSs was calculated for determining the crystalline and amorphous phases and the values are shown in Table 6.

**Table 6:** Crystalline and amorphous phase values of EPS calculated using crystalline index from XRD analysis data (values in percentage).

EPS	Crystalline phase	Amorphous phase
EPS-Psh	24	76
EPS-Vha	22	88
EPS-Pla	23	77

### SEM analysis of EPS

**Figure 8:**
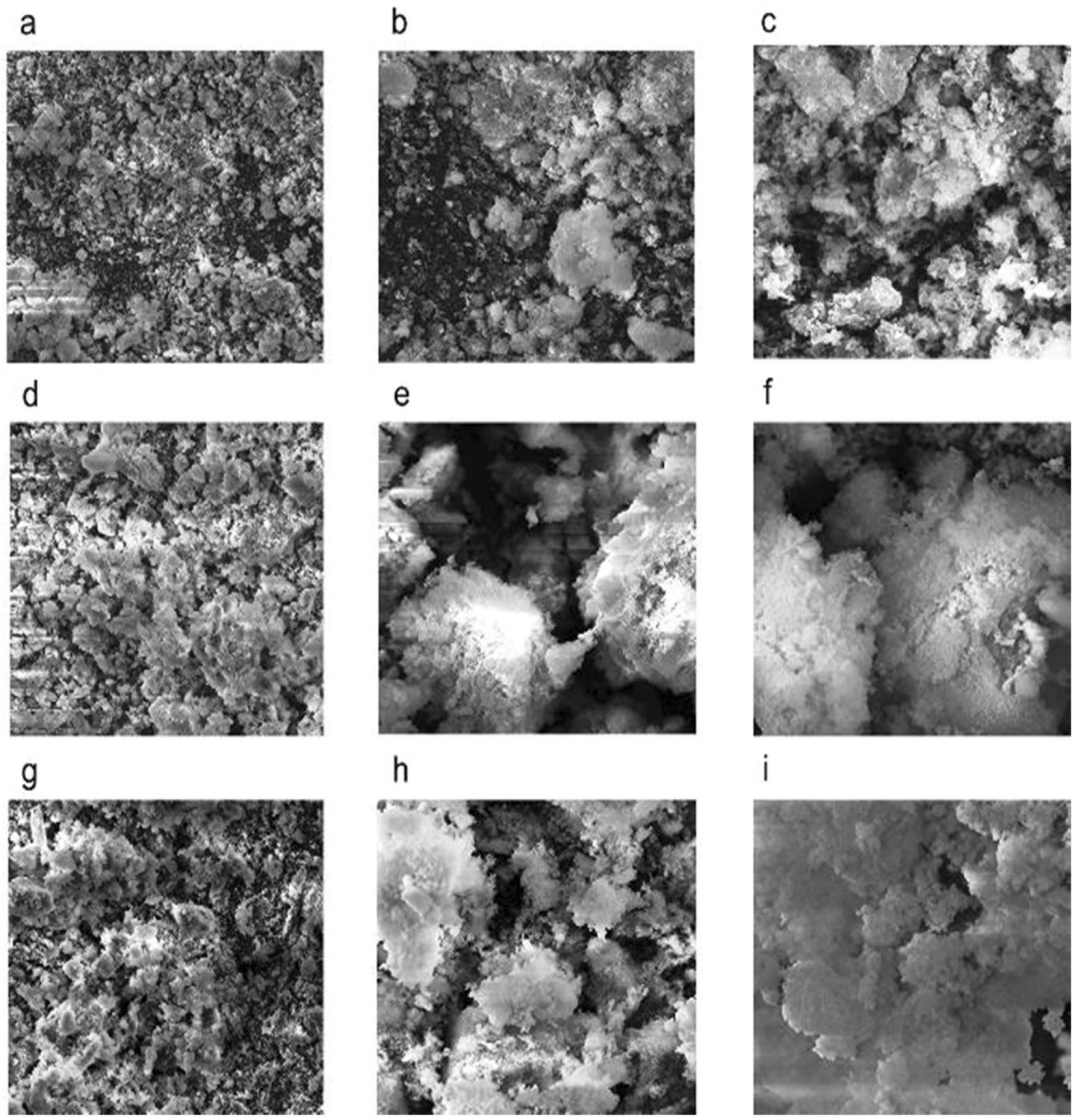
Scanning electron microscopy image of EPS isolated from *Pseudoalteromonas shioyasakiensis* (**a–c**), *Vibrio harveyi* (**d–f**) and *Planomicrobium* sp. (**g–i**).

The SEM images of three EPSs clearly described the compact nature of exopolymers (Fig. 8). There was no visible difference in the morphology of all the three EPSs. The exopolymers obtained from *P. shioyasakiensis, V. harveyi* and *Planomicrobium* sp. consisted of aggregated and irregular sphere-shaped particles under 5.0 kx magnification.

## Discussion

The settlement assay results of the present study indicated the importance of water-soluble cues as settlement inducers for barnacle larvae. The EPSs isolated from *P. shioyasakiensis*, *V. harveyi* and *Planomicrobium* sp. showed better settlement induction rate compared with the control. However, the percentage of larval settlement induced by EPS-Pla was high when compared with EPS-Psh and EPS-Vha (Fig. 1). This indicates that the larval settlement-inducing activity of the exopolymers may differ with the source microbial species^31^. The present results are consistent with previous work that barnacle cyprids depend on water-soluble polymeric cues for larval response due to the absence of substratum-coated cues^27,31^.

The active substrate selection and settlement of marine invertebrates are induced by cues believed to be chemical in nature^28^. The chemical cues responsible for settlement has been recognized by the chemoreceptors of marine invertebrate larvae^32,33^. Earlier, the role of chemical cues for settlement was reported only for few marine invertebrate larvae, including mud snail *Ilyanassa obsoleta*^34^, sand dollar *Dendraster excentricus*^35^, coral-eating nudibranch *Phestilla*^36^ and the oyster *Crassostrea gigas*^37^. Induction of larval settlement by bacteria-derived metabolites has been well documented in the literature^23^. For instance, water-soluble exopolymers and bacterial metabolites induced settlement of the tube worm *H. elegans*^25^.

^1^H NMR analysis is a powerful tool for chemical fingerprinting and in this study, polymeric samples from different sources were analysed for the presence of different types of chemicals^38^. Results revealed chemical shifts indicating the presence of sugar ring protons, proteins and acetyl groups (Fig. 3). An anomeric proton region in the NMR spectra was revealed through the presence of proton signals between 4.1 and 5.4 ppm^39^. The peaks around 2.2 ppm spectra have been assigned to acetyl amines of hexose or pentose^40^. The chemical shift at 3.4 ppm was attributed to exopolymers, proteins, acetyl and succinyl groups^41^. The proton signals at 4.1 ppm were attributed to fucosylated compounds in the EPS samples^42^. The spectral shift at 2.0, 4.4 and 4.6 ppm showed the presence of *N*-acetyl and *N*-acetyllactosamine^43^. The chemical shift at 5.3 ppm was ascribed to tetraose compounds^44^. The proton signals between 3.5 and 4.0 ppm were common in all the three exopolymeric samples. The chemical shift in the particular region was ascribed to N-glycans and linked to mannose compounds^45^.

The biochemical characterization of exopolymers showed that the polysaccharide was the major component and the protein content was lower than the total carbohydrates (Table 2). GC-MS analysis of the glycosyl composition of the exopolymers produced by EPS-Psh, EPS-Vha and EPS-Pla were composed of N-glycans, fucosylated compounds and oligomannose glycans, *N*-acetyllactosamine (Tables 3–5). The glycans has a complementary function in governing the strength and viscosity of a biofilm matrix produced by bacteria^46^. Previous research has shown that N-glycans, fucosylated oligomannose and oligomannose glycan moiety induce the settlement of *A. amphitrite* cyprids^47,48^. *N*-acetyllactosamine identified in our study has been reported earlier for its settlement-inducing activity of coral larvae *Acropora* spp. and *Agaricia humilis*^23,24^. Earlier it has been identified that exogenous polysaccharides induced larval settlement and metamorphosis through mannose-binding receptors in spirorbids, ascidians and corals^49–51^. Furthermore, transcriptomics and proteomics studies revealed the presence of Malectin-A and mannose-binding receptors in *A. amphitrite* cyprid stage^52,53^. Therefore, the results of this study suggest that N-glycans and oligomannose-containing exogenous polysaccharides could bind to the mannose-binding receptors of larvae and induce larval attachment.

FT-IR functional group analysis of crude EPS indicated the presence of carbohydrates, pyranose, mannose and uronic acids (Fig. 6). The presence of these compounds in microbial EPS has been reported previously^54–57^. The peak around 3407–3414 cm^−1^ and 2926–2929 cm^−1^ correspond to hydroxyl and CH_2_ groups respectively^58,59^. The peak around 1421–1424 cm^−1^ indicated the presence of a –COO^−^ group^40^. Further, the peaks at around 1127–1147 cm^−1^ showed the presence of pyranose, uronic acid and o-acetyl ester linkage bonds^54,57^. The absorption peak around 500–650 cm^−1^ was attributed to the presence of pyranose rings^56^.

XRD analysis revealed the crystalline nature of polymers. EPSs were mostly amorphous in nature, which was reported previously for *Vibrio parahaemolyticus*, *Bacillus licheniformis* B22 and *Scenedesmus* sp. SB1^40,60,61^. Since very few pieces of evidence are available for the X-ray diffraction analysis of exopolymers, results obtained in this study will further enhance our knowledge of the properties of exopolymers isolated from marine biofilms. The SEM analysis is commonly used to study the surface morphology of exopolymers. The surface features of EPS isolated in the present study exhibit similar morphological characteristics of irregular and aggregated particles as described earlier^62^.

In conclusion, the results of this study indicated the ecological role of *N*-acetyl, N-glycans and oligomannose present in exopolymers of marine bacteria. Further, the compounds identified from the marine bacterial exopolymers were similar to compounds reported from the settlement-inducing protein complex (SIPC) of *A. amphitrite* and coralline algal cell wall polysaccharides. Hence, the present study suggests that the cues for larval settlement may possess some kind of similarity in chemical composition irrespective of origin. Further research focusing on the structural characterization of biofilm exopolymers and larval binding receptors will provide more insights into the larval settlement process on hard substrates in marine ecosystems.

## Materials and Methods

### Barnacle larval rearing for settlement assay

Adult barnacles (*A. amphitrite*) attached to rocky substrates were collected from the Obhur Creek near the King Abdulaziz University marine station. Adult organisms along with the substrate were maintained in seawater at an ambient temperature (28 °C). The barnacles were exposed to air for less than an hour to induce nauplii release. The nauplii released were collected using a hand net (80 µm) and maintained in a 2 L polypropylene aquarium containing 0.7 µm filtered seawater (FSW). The larvae were fed *Chaetoceros calcitrans* at a density of 2 × 10^5^ cells ml^−1^. The photoperiod of 12:12 h light and the dark cycle were maintained during cyprid culture. Water was changed daily and renewed with fresh microalgal diet. Nauplii stages were checked under a stereomicroscope (Leica S6E) until the cyprid stage was noticed (5–6 days). Cyprids were collected from the aquarium using a Pasteur pipette and used for settlement studies.

### Extraction and purification of exopolymers

Biofilm-forming bacteria were isolated from natural biofilms that developed on aquaculture cage nets submerged in the Obhur Creek, Central Red Sea. *P. shioyasakiensis* (NCBI GenBank accession number: KY224086), *V. harveyi* (NCBI GenBank accession number: KY266820) and *Planomicrobium* sp. (NCBI GenBank accession number: KY224087) were the dominant biofilm bacteria isolated from the cage nets^63^. EPSs were extracted from the marine bacteria by following the method of Zhang *et al*.^64^ with few modifications. The bacteria were cultured in modified seawater nutrient broth (peptone 10 g, beef extract 10 g, sodium chloride 5 g, 0.2 µm FSW 1 L) at an ambient temperature of 25 °C for 2 days. The bacterial cultures were centrifuged at 10000 × *g* at 4 °C for 20 min. The bacterial pellets were removed and the supernatants were filtered through nitrocellulose membrane filter (0.45 µm). The filtered supernatants were used for EPS extraction. EPSs were precipitated by adding equal volume of ice-cold absolute ethanol and refrigerated at 4 °C for 48 h. The precipitated crude EPSs were collected from the medium and washed with double distilled water and freeze-dried. The freeze-dried crude EPSs were purified using dialysis membrane (12–14 kDa cut-off). The crude EPSs were dialysed against double distilled water overnight. The purified EPSs were freeze-dried and used for characterization and bioassays. The isolated EPSs were then named as EPS-Psh (*P. shioyasakiensis*), EPS-Vha (*V. harveyi*) and EPS-Pla (*Planomicrobium* sp.). The total carbohydrate content of the EPSs was determined by the phenol-sulphuric acid assay using d-glucose as a standard solution^65^. The total protein content was measured by using the protein quantification assay (Roti-Quant, Carl Roth GmbH) according to the methods described by Bradford^66^ (1976). Bovine serum albumin (99.8%) crystals were used as a protein standard and the assay was carried out in 96-microwell culture plates^67^.

### Fluorescence microscopy imaging of biofilm

In order to confirm the biofilm-forming ability of the bacteria strains, the biofilms of *P. shioyasakiensis, V. harveyi* and *Planomicrobium* sp. were grown in gamma-sterilized crystal grade polystyrene 24-well cell culture plates (SPL Life Sciences) for 24 h under static conditions at 28 °C. After 24 h, the broth was removed and the wells were slightly rinsed with 0.2 µm FSW to remove planktonic bacterial cells. For nucleus staining, the biofilm bacterial cells were stained with 50 µl of 0.01% DAPI solution (4′,6-diamidino-2-phenylindole) and incubated for 5 min in the dark. After incubation, the wells were washed with 1x PBS and dried before imaging. Images were collected using fluorescence microscope EVOS FL Cell Imaging System (Thermo Fisher Scientific, CA) equipped with RFP (red fluorescence), GFP (green fluorescence) and DAPI light cubes.

### Larval toxicity assay

The EPS toxicity to barnacle larvae was assessed by treating the EPS suspension against stage III nauplii. The EPS (EPS-Psh, EPS-Vha and EPS-Pla) stock solutions were prepared in FSW at a concentration of 10 mg ml^−1^. The assay was performed by adding 10 nauplii to 12-well plates containing 3 ml of different EPS concentrations (0, 250, 500, 750 µg L^−1^) in 0.2 µm FSW. The experiment was carried out (in replicates, n = 4) for 48 h in a climate-controlled walk-in environmental chamber at an ambient temperature of 28 °C.

### Larval settlement assay

Barnacle larval settlement assay was performed by adding 10 cyprids to six-well plates (Falcon) containing 10 ml of different EPS concentrations (0, 250, 500, 750 µg L^−1^) in 0.2 µm FSW. The experiment was carried out (in replicates, n = 6) for 48 h in a climate-controlled walk-in environmental chamber at an ambient temperature of 28 °C. After 24 and 48 h, the number of settled and metamorphosed cyprids in the multi-well plates was checked under a stereomicroscope (Leica S6E) (Supplementary Figure S1). Data obtained from larval settlement assay were subjected to two-way ANOVA to understand the variations between treatments. Concentration of EPS and larval settlement observation time (24 and 48 h) were used as factors. For statistical tests, P < 0.05 was considered as significant. Also, the EC_50_ values (the concentration in which 50% settlement induction was observed) were calculated based on regression analysis between the EPS concentration and percentage of settlement observed.

### ^1^H NMR analysis

For proton NMR analysis, 30 mg of lyophilized EPS samples was dissolved in 500 µl of deuterated DMSO and kept in a 5 mm NMR tube. The NMR spectra were recorded at 300 K using a Bruker AVANCE III 600 MHz equipped with a CryoProbe. The chemical shifts were expressed in parts per million (ppm) relative to internal chemical shift reference, 4-dimethyl-4-silapentane-1-sulfonic acid. The NMR peaks obtained were integrated and picked by Bruker Topspin 3.2 software.

### GC-MS analysis

Glycosyl composition of the exopolymers from three marine biofilm bacteria was carried out as described by Hammi *et al*.^68^ with little modifications. The purified EPSs (4 mg) added with 50 *μ*L of myo-inositol (2 mg mL^−1^, internal standard) were hydrolysed by 2 M trifluoroacetic acid at 90 °C for 2 h in a sealed glass tube. Then, the hydrolysed lysates were freeze-dried. Trimethylsilyl derivatives were prepared by adding 150 μL of pyridine and 150 μL of *N*, O-bis trimethylsilyl trifluoroacetamide to the dried samples. The samples were heated to 80 °C for 30 min and then evaporated to dryness. The residues were dissolved in 750 μL of dichloromethane and trimethylsilyl derivatives were analysed by a 7000 C triple quadrupole gas chromatography-mass spectrometry system (Agilent Technologies). The instrument was equipped with a high-sensitivity extractor ion source and a triple-axis HED-EM detector which can identify trace components even in complex mixtures at the femtogram level. For statistical tests, the samples were analysed by GC-MS in triplicate. The triplicate samples of EPS-Psh were labelled as Psh1, Psh2 and Psh3. Similarly, the EPS-Vha and EPS-Pla samples were named as Vha1, Vha2, Vha3 and Pla1, Pla2, Pla3 respectively.

The Agilent MassHunter, Openchrom (Dalton, Lablicate and Scientific community), Automated Mass Spectral Deconvolution and Identification System (AMDIS v2.73), NIST MS search, NIST glycan MS library (National Institute of Standards and Technology, USA), MetaboAnalyst 4.0 were used for mass spectral acquisition, peak processing, spectral deconvolution, compound identification, data visualization and statistical analysis. GC-MS spectra for the exopolymers samples were acquired using an Agilent MassHunter software and the data were exported (.mzdata.xml file) to OpenChrom mass spectral analysis software for peak integration and active peak identification. The MS peaks were deconvoluted using AMDIS software; the retention indices were searched against NIST MS library and NIST glycan MS library search for compound identification. The compounds from the top hit list of the library search were reported. The statistical analysis for the MS peak list and intensity data were performed using MetaboAnalyst 4.0 software. The MS peak list data were uploaded as a CSV (.csv) file; a further quality check of data and normalization by autoscaling were done. The normalized data were used for PCA and correlation heatmap analysis.

### FT-IR analysis

All the three EPSs were subjected to functional group analysis using Fourier-transform infrared (FT-IR) spectroscopy. Three milligrams of lyophilized EPS samples was pressed in KBr pellets and the spectral wavelength was recorded in the range of 4000–400 cm^−1^ with a resolution of 4 cm^−1^ using an FT-IR spectrometer (Perkin Elmer)^69^. The FT-IR were analysed and the functional groups were interpreted using KnowItAll(R) Informatics System from Bio-Rad Laboratories. The FT-IR spectra of all the EPSs were stacked together to elucidate similarities in spectral regions.

### X-ray diffraction analysis of EPSs

Powder XRD analysis is extensively used for the characterization of polymers and is useful for studying the crystalline nature of exopolymers derived from different origins^68^. EPSs (EPS-Psh, EPS-Vha and EPS-Pla) were analysed by XRD using a multipurpose X-ray diffraction system (Rigaku Ultima IV XRD) with 2Θ ranging from 2 to 80° at 25 °C. The irradiated length and specimen length were 10 mm, with a receiving slit size of 0.2 mm at a 285 mm goniometer radius. The distance from the focus to divergence slit was 100 mm. Lyophilized EPS samples were mounted on quartz substrata, and the intensity peaks of diffracted X-rays were continuously recorded with a scan step time of 1 s; d-spacings appropriate to the diffracted X-rays at each Θ value were calculated from Bragg’s law (Eq. 1).

1 $$d=\lambda /2\,\sin \,\Theta $$

where

*d* is the interplanar distance,

*λ* is the incident wavelength and

Θ is the scattering angle measured from the incident beam.

The crystallinity index (CI_xrd_) was calculated as the ratio of the crystalline phase peak areas to the sum of the crystalline peak areas and the amorphous profile (Eq. 2)^70^.

2 $${{\rm{CI}}}_{{\rm{xrd}}}=\Sigma {A}_{{\rm{crystal}}}/{\Sigma A}_{{\rm{crystal}}}+\Sigma {A}_{{\rm{amorphous}}}$$

### SEM analysis of EPS

The surface morphology of EPSs was identified using scanning electron microscopy (VEGA3 TESCAN) with an accelerating voltage of 20 kV and 10 mm working distance. Lyophilized EPS samples were mounted on the metal and gold sputtered prior to imaging. SEM micrographs were captured at 500x, 2.0 kx and 5.0 kx magnification^71^.

## Supplementary information

Supplementary figures

## Data Availability

All generated data used for the analysis are available upon request from the corresponding author.
