# Supplementary material for: Characterization and assessment of barnacle larval settlement-inducing activity of extracellular polymeric substances isolated from marine biofilm bacteria
Source: Sci Rep. 2019 Nov 28;9:17849. doi: 10.1038/s41598-019-54294-9 (PMC6882797; doi:10.1038/s41598-019-54294-9)
Supplement: Supplementary file 1 — Supplementary figures [file 41598_2019_54294_MOESM1_ESM.docx]

**Supplementary materials for:**

**Characterization and assessment of barnacle larval settlement inducing activity of extracellular polymeric substances isolated from marine biofilm bacteria**

**Aboobucker Siddik, Sathianeson Satheesh***

**Department of Marine Biology, Faculty of Marine Sciences, King Abdulaziz University, Jeddah, Saudi Arabia**

**E-mail: ssathianeson@kau.edu.sa, satheesh_s2005@yahoo.co.in**


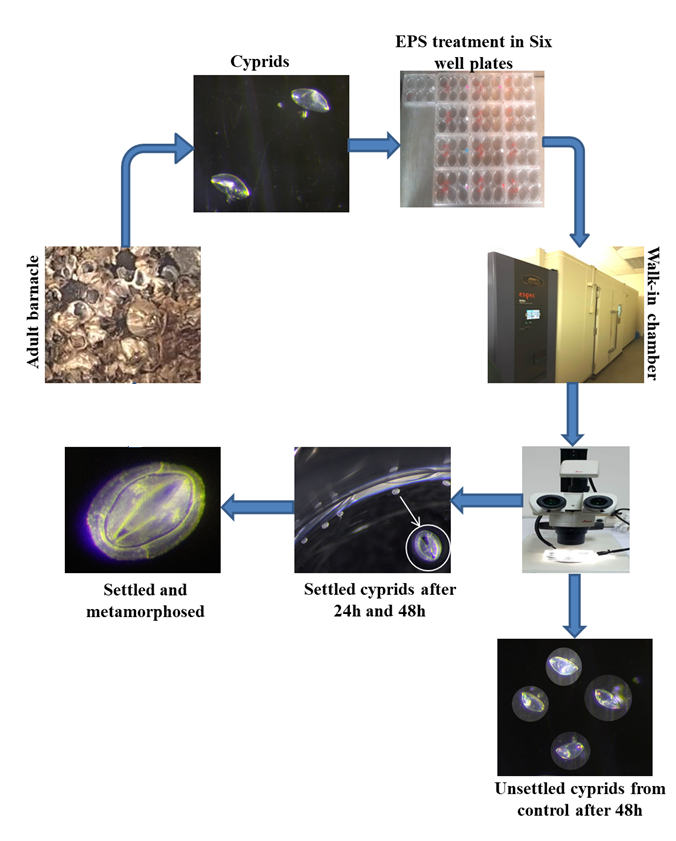


Figure S1. Assessment of barnacle larval settlement inducing activity of EPSs


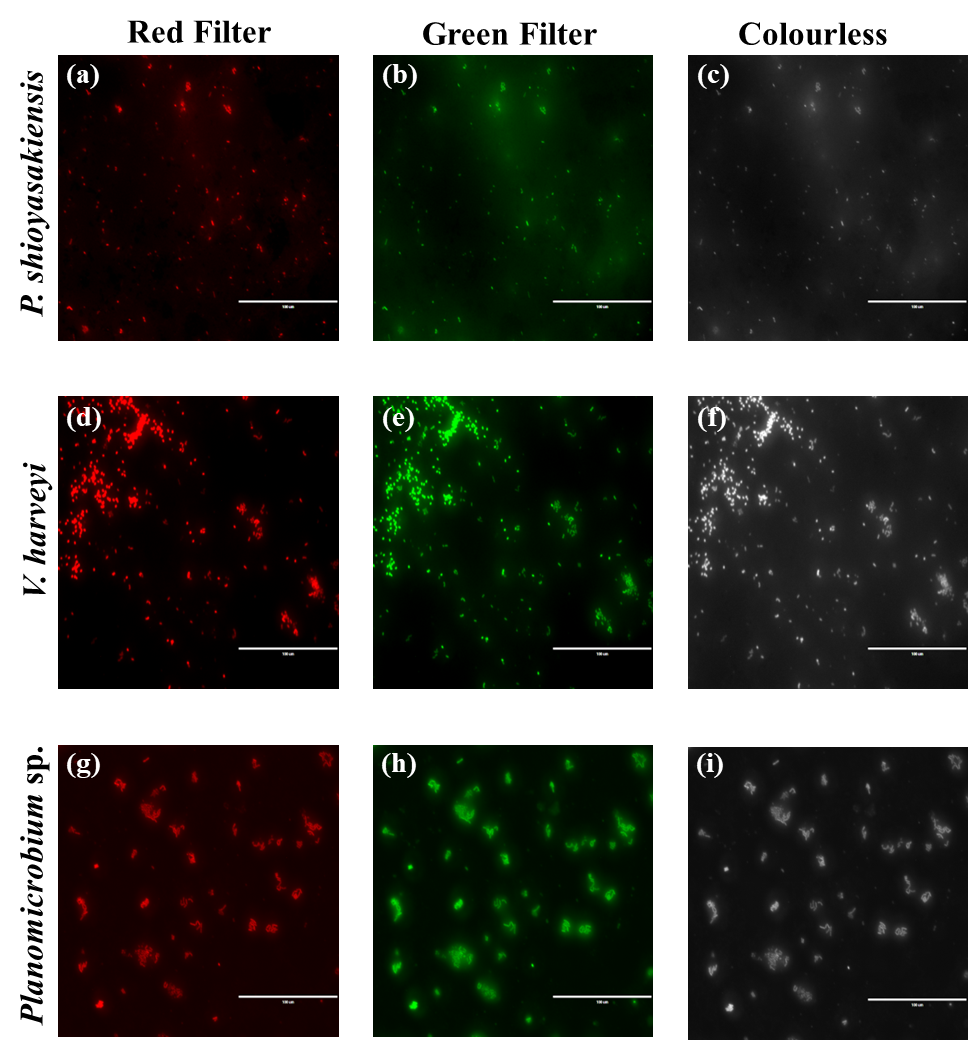


Figure S2. Fluorescence microscopy visualization of biofilms developed on 24-well cell culture plates (scale 100 𝜇m): (a-c) *P. shioyasakiensis*, (d-f) *V. harveyi* and (g-i) *Planomicrobium* sp.
